# The effects of high-tannin leaf litter from transgenic poplars on microbial communities in microcosm soils

**DOI:** 10.3389/fmicb.2013.00290

**Published:** 2013-09-26

**Authors:** Richard S. Winder, Josyanne Lamarche, C. Peter Constabel, Richard C. Hamelin

**Affiliations:** ^1^Pacific Forestry Centre, Canadian Forest Service, Natural Resources CanadaVictoria, BC, Canada; ^2^Laurentian Forestry Centre, Canadian Forest Service, Natural Resources CanadaSainte-Foy, QC, Canada; ^3^Centre for Forest Biology, University of VictoriaVictoria, BC, Canada

**Keywords:** transgenic, tree, forestry, risk assessment, *Populus tremuloides*, nitrogen cycle

## Abstract

The impacts of leaf litter from genetically modified hybrid poplar accumulating high levels of condensed tannins (proanthocyanidins) were examined in soil microcosms consisting of moss growing on sieved soil. Moss preferentially proliferated in microcosms with lower tannin content; DGGE (denaturing gradient gel electrophoresis) detected increased fungal diversity in microcosms with low-tannin litter. The proportion of cloned rDNA sequences from Actinobacteria decreased with litter addition while Bacteroidetes, Chloroflexi, Cyanobacteria, and α-Proteobacteria significantly increased. β-Proteobacteria were proportionally more numerous at high-tannin levels. Tannins had no significant impact on overall diversity of bacterial communities analyzed with various estimators. There was an increased proportion of N-fixing bacteria corresponding to the addition of litter with low-tannin levels. The addition of litter increased the proportion of Ascomycota/Basidiomycota. Dothideomycetes, Pucciniomycetes, and Tremellomycetes also increased and Agaricomycetes decreased. Agaricomycetes and Sordariomycetes were significantly more abundant in controls, whereas Pucciniomycetes increased in soil with litter from transformed trees (*P *= 0.051). Richness estimators and diversity indices revealed no significant difference in the composition of fungal communities; PCoA (principal coordinate analyses) partitioned the fungal communities into three groups: (i) those with higher amounts of added tannin from both transformed and untransformed treatments, (ii) those corresponding to soils without litter, and (iii) those corresponding to microcosms with litter added from trees transformed only with a β-glucuronidase control vector. While the litter from transformed poplars had significant effects on soil microbe communities, the observed impacts reflected known impacts on soil processes associated with tannins, and were similar to changes that would be expected from natural variation in tannin levels.

## INTRODUCTION

With increasing global demand for wood products, interest has grown in methods that could potentially provide trees with promising new and desirable traits for the forest products industry. Using genetic modification to enhance valuable traits in trees without compromising desirable genetic background offers an alternative to conventional breeding in this context, by permitting the transfer of genetic material into selected genotypes. Exogenous genes, transferred from unrelated organisms, can confer novel traits such as herbicide, disease, or pest resistance. Research using transgenic methods has been conducted on more than thirty tree species (Peña and Seguin, 2001; Gartland et al., 2003; van Frankenhuyzen and Beardmore, 2004). Poplar is considered a model tree for transgenic transformation because of the wealth of extant information on its genomics (Jansson and Douglas, 2007), its susceptibility to genetic transformation (Morse et al., 2004), and its rapid growth, making it amenable for use by for the pulp and paper industry (Stanton et al., 2002).

Concerns about potential risks associated with out-planting genetically modified trees on the landscape have led to regulatory restrictions (bans) on their use in Canada (Hay et al., 2002; van Frankenhuyzen and Beardmore, 2004). While the use of genetically modified trees may not be an immediate human health risk, concerns do arise about the potential impacts of these trees within ecological systems. For example, it has been suggested that transgenic trees might accelerate the development of pest resistance and give rise to new pest dynamics (Hay et al., 2002; Marvier, 2002). Other concerns include the possibility of gene escape (Hay et al., 2002) and the potential for “genetic pollution” of natural populations. These issues are pertinent to the biodiversity of native forest ecosystems, which is considered more valuable than the biodiversity of agro-ecosystems because trees are a particularly long-lived component (Gartland et al., 2003; Halpin et al., 2007). Thus, it is important to pay particular attention to the potential “side-effects” that transgenic trees such as poplar might have on their immediate environment, including the soil environment and its key nutrient cycling microbial communities.

Laboratory, greenhouse, and field studies have largely reported little to no impact of transformation on microbial communities of soils (Hampp et al., 1996; Kaldorf et al., 2002; Pasonen et al., 2005; Lamarche and Hamelin, 2007; Newhouse et al., 2007; Seppänen et al., 2007; Oliver et al., 2008; Stefani et al., 2009, 2010; Lottman et al., 2010; Lamarche et al., 2011), although some have found significant changes in particular microbial groups (LeBlanc et al., 2007; Andreote et al., 2009). These responses to genetic modifications occur within a wider context of variable natural genotypic influences on microbial communities. For example, in common garden experiments comparing naturally occurring genotypes of *Populus angustifolia* and *Populus fremontii*, heritable genotypic variation was found to cause up to 75% of the variation in microbial biomass and 70% of the variation in community composition (Schweitzer et al., 2008).

Although leaf litter is a prime avenue through which trees may impact their soil environment, there has been limited study focused on the environmental impacts of litter from transgenic forest trees. In one field study, Vauramo et al. (2006) found that fungal biomass was similar in leaf litter from controls versus that from silver birch trees transformed with a chitinase gene. Given the ecological importance of leaf litter, much further work is needed to delineate the potential impacts of litter from transformed trees in natural forests and forestry situations.

This study addresses the potential impacts of leaf litter from transgenic hybrid poplar (*Populus tremula* × *tremuloides*) that accumulate high concentrations of proanthocyanidins (condensed tannin) due to over-expression of the PtMYB134 transcriptional regulator (Mellway et al., 2009). Depending on growth conditions, these transgenics can accumulate 5–50-fold higher levels of leaf tannins compared to control plants. The tannins found in the leaf litter of poplars significantly influence decomposition and other soil processes (Mansfield, 1999; Schweitzer et al., 2004; Madritch et al., 2006). Tannin levels in poplar are variable but genetically determined, and are implicated in diverse ecological adaptations including defense against pests and pathogens, as well as other stress responses (Witham et al., 2006; Constabel and Lindroth, 2010). Many tannins have antimicrobial properties (Scalbert, 1991); in poplar, they may defend against fungal pathogens (Miranda et al., 2007; Holeski et al., 2009). In general, tannins are reported to have a number of potential modes of action against microbes, including inhibition of microbial binding to biomaterials (Howell et al., 1998), and inhibition of enzymatic activity (e.g., Adamczyk et al., 2009). Tannins found in leaf litter are associated with reduced activity of hydrolases (β-glucosidase, *N*-acetyl-glucosaminidase), peroxidase, and acid phosphatase in soils (Joanisse et al., 2007; Triebwasser et al., 2012) and decrease net N-mineralization and gross ammonification while increasing N-immobilization (e.g., Bradley et al., 2000); proanthocyanins and other condensed tannins were observed to be no less labile or inhibitory than hydrolysable tannins (Kraus et al., 2004). In *Populus balsamifera* growing in the Alaskan taiga, Schimel et al. (1998) attributed major changes in soil N-cycling to the secondary compounds found in poplar leaves; poplar litter reduced N-fixation, decomposition, and N-mineralization, while increasing N-immobilization. Some reports indicate that this inhibition of N-cycling can be qualified by the molecular weight of condensed tannins, the vegetative history of the soil, and by the microbial community present (Fierer et al., 2001; Triebwasser et al., 2012); however, research in other types of plant litter has found some of these influences to be inconsistent (Norris et al., 2011).

The general objective of this study was to develop a model microcosm system for testing impacts of transgenic litter in microcosms, as a tool for both risk analysis and environmental modeling. Microcosms are reported to be useful in assessing the effects or potential risks associated with genetically modified organisms on a smaller scale (Bolton et al., 1991; Krimsky et al., 1995; Pasonen et al., 2005), and the effects of leaf litter on nutrient dynamics (Madritch and Hunter, 2003).

Our specific goal was to study the impacts of leaf litter from genetically modified hybrid poplar accumulating high proanthocyanidin levels on microbial communities in very simplified microcosm soils supporting live moss. In the absence of extensive roots, mycorrhizal networks, and mobile organisms, microbial populations in the microcosms were restricted to immediately adjacent nutrient resources. This simplification was performed in order to increase resolution for baseline processes and limit the fluctuation of C and potentially confounding effects from nutrient subsidies (Teuben and Verhoef, 1992; Machinet et al., 2009; McGuire and Treseder, 2009). We hypothesized that the microbial communities associated with litter from poplar with high-tannin levels would be significantly different in terms of both structure and abundance of predominant species or groups. We also predicted that these changes would reflect the known impacts on soil processes associated with tannins.

## MATERIALS AND METHODS

### TRANSGENIC POPLAR LITTER

Transgenic poplars used in this study were hybrid poplar (*Populus tremula* × *tremuloides*) over-expressing the PtMYB134 proanthocyanidin regulator under control of the double 35S promoter, described by Mellway et al. (2009). Control plants consisted of the parental genotype (clone INRA 353-38; Leple et al., 1995) and plants over-expressing a β-glucuronidase reporter (GUS vector; GV) gene as a transgenic control (Mellway et al., 2009). Leaf litter from two typical transgenic lines expressing high levels of proanthocyanidin (HP lines: MYB 134-62 and MYB 134-48), the GV line (GUS 41), and the parental wild-type line (PT) was collected as leaves senesced and abscised from 1.5-m saplings. Leaves were stored in darkness at 20°C. In order to acquire sufficient litter for the experiment, equal amounts of leaves collected from the two HP lines were combined into one pooled lot.

### SOIL COLLECTION AND MICROCOSM ASSEMBLY

Soil was collected in November 2006 from a mature plantation of non-transgenic *Populus trichocarpa* and *Populus trichocarpa* × *deltoides* [TxD] hybrid clones (16 year-old) located at the Scott Paper Nursery, Harrison Mills, BC, Canada within an area designated “Espacement Trial 1988.” The soil at this location is acidic (pH 5.6) and alluvial, classified as Monroe Clay Loam, with organic matter and N comparable to upland soils, concentration of P and Ca in the A_0_ horizon, and other elements evenly distributed (Kelley and Spilsbury, 1939). Prior to the plantation, the area was used to pasture cows. The understorey grass at the plantation was previously cut several times, with the last mowing occurring 2 years prior to soil sampling. Within the espacement trial, poplar clones (*Populus trichocarpa* var. “Blom”, clones “B.C.,” “TxD 49-177,” “TxD 50-197,” and “TxD 44-143”; Quinsey et al., 1991) had been replicated along transects with spacing treatments ranging from 3.6 to 4.8 m. The *Populus trichocarpa* clones were derived from poplars indigenous to nearby Chilliwack, B.C. Soil samples were taken using a trowel to excavate ca. 10 cm^2^ to a depth of ca. 5 cm. The samples were acquired from plots corresponding to four different clones, with rows of trees spaced 4.5 m apart. The understorey vegetation primarily consisted of various grass species. In each plot, five samples were taken at random points, midway between the rows. Each soil sample was passed through a sieve (4 mm) and an equal portion from each was added to a single pooled lot; the lot was manually homogenized to form a single composite soil. Moisture of the composite soil was adjusted to ca. 15% through the addition of dH_2_O and then divided into four portions corresponding to poplar litter derived from the three different sources (HP, GV, PT) plus a soil control.

A completely randomized experimental design was employed to assay the soil in microcosms. Leaf lots were separately mulched through a 4-mm sieve, and litter was added to three separate lots of the composite soil (1.3% w:w) and thoroughly mixed by hand to create a soil–litter mix. Microcosms (three replications per leaf litter type) were constructed by adding ca. 100 g of soil–litter mix to a sterile 250-mL Erlenmeyer flask, plugging the flask with a foam cap, and covering the flask with aluminum foil; all steps were performed using aseptic technique. Prior to sealing the microcosms, 0.5 g of soil–litter mix was removed from each 100-g replication, pooled to form a non-incubated control, and stored at -20°C for subsequent analysis of initial microbial communities. The microcosms were weighed and placed in random, assigned positions in a growth chamber within a 3 × 4 grid spaced ca. 10 cm apart. The microcosms were incubated at 20°C with a 16-h diurnal photoperiod for 60 d. Microcosms were weighed daily, and sterile water was added to each flask with a sterile pipette to maintain the initial flask weight. Germinating seedlings of any plants other than mosses were removed with sterile forceps. At the end of incubation, the proliferation of mosses in each microcosm was visually estimated, as the differences in moss growth were readily apparent. Moss growth was ranked on a scale of 1–4, where “1” corresponded to less than 5% coverage of the soil surface, “2” corresponded to 5–33% coverage of the soil surface, “3” corresponded to 33–90% coverage of the soil surface, and “4” corresponded to complete coverage of the soil surface. There was no variance in the results, therefore they were excluded from statistical analysis.

After incubation, soil–litter mix was stored at 4°C prior to subsequent analysis. For each soil/litter lot, the initial proanthocyanidin content of leaves (GV: 22.65 mg g^-^^1^; PT: 45.45 mg g^-^^1^; HP: 109.95 mg g^-^^1^) was confirmed by extraction with 80% aqueous MeOH and assaying tannins according to the BuOH–HCl method of Porter et al. (1986). Purified tannin from *Populus tremuloides* was used as a standard.

### DNA EXTRACTION

After an incubation period of 60 d, duplicate 0.15-g soil–litter mix samples were removed from each microcosm and each control lot with a sterile metal scoop. The samples were extracted using UltraClean^®^ Soil DNA kits (MoBio Laboratories, Carlsbad, CA, USA), according to manufacture’s instructions. To reduce the PCR inhibition noted in preliminary trials, the isolated DNA was precipitated by adding a mixture of 0.1 volume of 7.5 M NH_4_OAc buffer and 30 μg (6 μl)of Co-precipitate (Bio-Line USA Inc., Taunton, MA, USA), and 2 volumes of cold 95% EtOH to the samples. The samples were incubated at room temperature for at least 30 min prior to being centrifuged at 13,000 × *g*. Precipitated pellets were washed with 70% EtOH, air-dried, and re-suspended in TE buffer, pH 8.0. Extracted DNA was visualized through electrophoresis with a 1% agarose EtBr gel and imaged using a transillumination system (Chemigenius Q with GeneSnap software, Syngene USA, Frederick, MD, USA). Duplicate DNA samples were pooled and quantified using a spectrophotometer (Nanodrop-1000, Nanodrop Technologies, Wilmington, DE, USA).

### PCR-DGGE

A preliminary check of DNA diversity was performed with one sample from each microcosm after 30 and 60 d incubation, using PCR-DGGE. The primers used to amplify 16S and 18S rDNA regions are listed in **Table 1**. All incubations and PCR steps were performed in a T-gradient thermocycler (Biometra GmbH, Goettingen, Germany). Prior to PCR steps, samples were heated at 96°C for 3 min. and then cooled to 80°C before addition of 5 μl of Master Mix with Gold TAQ (Invitrogen Canada Inc., Burlington, ON, Canada). For 16S rDNA amplification, PCR was performed according to the protocol of Fortin et al. (2004), while PCR for 18S rDNA was performed according to the protocol of Vainio et al. (2005). PCR products were visualized by electrophoresis in a 1.8% agarose EtBr gel and imaging with the transillumination system. Denaturing gradient gel electrophoresis (DGGE) was performed using a D-Code^TM^ Universal Mutation Detection system (BioRad Laboratories Ltd., Mississauga, ON, Canada) according to the manufacturer’s instructions, with some modifications. PCR products were loaded onto an 8% (w/v) acrylamide/bisacrylamide (37:5:1) gel with a denaturing gradient ranging between 10 and 60% in 1× TAE buffer (40 mM Tris, 20 mM acetic acid and 1 mM EDTA at pH 8.3). Electrophoresis was continued for 3.5 h at 60°C and 200 V. Gels were stained using SYBR^®^ Gold (Invitrogen) and visualized using the transillumination system.

**Table 1:** Primers used to amplify 16S and 18S rRNA genes of microbes in soil microcosms.

Primer	Primer sequence	Assay	Reference
16S F: _341-357_* Forward	5′CCT ACG GGA GGC AGC AG P – 3′	PCR-DGGE	Fortin etal. (2004)
16S R: _758-740_ Reverse	5′ CTA CCA GGG TAT CTA ATC C – 3′		
			
18S FF390 Forward	5′ CGA TAA CGA ACG AGA CCT – 3′		Vainio etal. (2005)
18S FR1** Reverse	5′ AI CCA TTC AAT CGG TAI T – 3′		
			
16S M13 F2 Forward	5′ GTA AAA CGA CGG CCA GT – 3′	Clone library	Anonymous (2001)
16S M13 R2 Reverse	5′ AAC AGC TAT GAC CAT G – 3′		
			
18S nu-ssu-0817 Forward	5′ TTA GCA TGG ATT ATT RRA ATA GGA – 3′		Borneman and Hartin (2000)
18S FR1 Reverse	5′ AIC CAT TCA ATC GGT AIT – 3′		Vainio etal. (2005)

* For DGGE, the GC clamp added to the 5′ end of the primer was 5′ GCG GGC GGG GCG GGG GCA CGG GGG GCG CGG CGG GCG GGG CGG GGG 3′.

** For DGGE, the GC clamp added to the 5′ end of the primer was 5′ CCC CCG CCG CGC GCG GCG GGC GGG GCG GGG GCA CGG GCC G3′.

### ISOLATION AND SEQUENCING OF rDNA CLONES

In order to determine the predominant 16S and 18S rDNA sequences present in the microcosm soils, rDNA clones were amplified and sequenced from the soil DNA extracts mentioned above. To ensure completion of sequencing within available resources, we restricted the analysis to treatments with added litter, and extracts from soil before litter addition and incubation. Extracts from microcosms incubated without litter were excluded from the analysis. Amplification of 16S rDNA from the DNA extracts was performed in 25 μl reaction volumes containing water, 1× buffer, 1.5 mM MgCl_2_, 200 μM dNTPs, 0.4 μM of each primers (**Table 1**), 1 U of Platinum *Taq* polymerase (Invitrogen), and 1 μl of DNA template. The reaction was performed in the T-gradient thermocycler (Biometra GmbH, Goettingen, Germany) with the following thermal protocol: initial denaturation of 94°C for 2 min, 35 cycles of 94°C for 1 min., 55°C for 30 s, 72°C for 1 min., and a final extension of 72°C for 5 min. Amplification of 18S rDNA from the DNA extracts was performed in 25 μl reaction volumes containing 1 mg/ml non-acetylated BSA, 1× buffer, 3 mM MgCl_2_, 200 μM dNTPs, 0.5 μM of each primers (**Table 1**), 1 U of Diamond DNA polymerase (Bioline), 1× Hi-Spec Additive (Bioline), and 1 μl of DNA template. The reaction was performed with the following thermal protocol: initial denaturation of 95°C for 2 min., 30 cycles of 94°C for 45 s, 55°C for 40 s, 57°C for 10 s, 72°C for 1 min., and a final extension of 72°C for 10 min.

The PCR products were cloned into vectors using the QIAGEN PCR Cloning Kit (Qiagen Inc., Valencia, CA, USA) according to the manufacturer’s protocol for 1,000 bp sequences (fivefold molar excess, 65 ng product, 30 min. incubation in ligation mixture). Successful transformants were picked, re-suspended in sterile dH_2_O, and boiled (at 100°C for 5 min) prior to PCR amplification. A total of 2304 clones were produced (twenty-four 96-well plates). Amplifications were performed using the same PCR process again, using 1 μl of boiled colonies as the DNA template. PCR products generating a single band of ~1210 bp (16S) or ~980 bp (18S) on the agarose gel were considered suitable candidates for sequencing. A total of 1,088 clones (448 for 16S rDNA and 640 for 18S rDNA) were submitted to McGill University and Genome Quebec Innovative Centre for sequencing, by single primer extension with the forward primer used in the PCR (**Table 1**). Samples that failed to provide a return sequence or returned sequences with a Phred quality score of less than 50 were excluded from analysis.

### BACTERIAL AND FUNGAL COMMUNITY ANALYSES

Sequences (16S and 18S rDNA datasets separately) were edited and assembled with Sequencher v4.6 (GeneCodes, Ann Arbor, MI, USA). Libraries were analyzed using Bellerophon (Huber et al., 2004) and potentially chimeric sequences were excluded from the data sets. The final 16S rDNA and 18S rDNA datasets included 430 and 622 sequences, respectively. Sequences were aligned with MUSCLE software v3.6 (Edgar, 2004). For each leaf litter treatment, rarefaction curves, Chao richness estimator as well as Shannon diversity index were computed with MOTHUR software v1.15.0 (Schloss et al., 2009) using 3 and 2% distance levels for 16S and 18S rDNA, respectively. Sequence alignments were converted in distance matrices using the DNADIST program (Jukes-Cantor as substitution model) from the PHYLIP package to produce neighbor-joining trees with MEGA version 4 (Tamura et al., 2007).

A phylum (bacteria) or class (fungi) classification was performed to identify major groups of microorganisms. For bacteria, we used the Ribosomal Database Project (RDP) Classifier tool to assign each sequence to a phylum using a naïve Bayesian rRNA classifier (Wang et al., 2007), with 80% similarity being the determining threshold. For fungi, consensus sequences of each operational taxonomic unit (OTU) were identified with the closest sequences found in the NCBI GenBank database using BLASTN (Altschul et al., 1990).

Separate principal coordinate analyses (PCoA) were performed for bacterial and fungal communities using UniFrac (accessed January 12, 2011; Lozupone and Knight, 2005). The PCoA used normalized abundance weights, treating each sample equally instead of treating each unit of branch equally.

### ANALYSES OF NUTRIENT CYCLING BACTERIAL GROUPS

Because proanthocyanidins affect N-cycling, phosphorus solubilization, and cellulose degradation, a more detailed taxonomic analysis was performed to categorize 16S rDNA sequences using matches to known or putative nitrogen-fixing or nitrifying microbes. Identities were ascribed to the sequences using BLASTN; the highest-ranking, most specific taxa at the 98–100% similarity level were used to designate sequences. This information was compared against the available literature to categorize organisms as N-fixing, nitrifying, phosphorus-solubilizing, or cellulose- (β glucoside-) degrading. Taxa higher than species were categorized with these functional attributes if a substantial majority of species were reported to possess the attribute.

### STATISTICAL ANALYSES

Analyses of variance for Chao richness estimator and Shannon diversity index were performed in R v2.9 (R Development Core Team, 2005) using the Vegan (R-Forge) package. The potential effects of leaf litter treatment and incubation time on the abundance of each bacterial phylum and fungal class were analyzed with a Poisson linear regression, performed with Statistica 8.0 © (Statsoft Inc., Tulsa, OK, USA). The data from the DGGE experiment and also from categorization of sequences pertaining to nutrient cyclers was also subjected to analysis of variance using Statistica 8.0, where Levene’s test was used to check for heterogeneity of variance and the Newman–Keuls test was used to determine significant differences. The proportion of nutrient cycling groups versus treatment was analyzed with the same software using multivariate analysis of variance (MANOVA) and the preceding tests for statistical assumptions.

### NUCLEOTIDE SEQUENCE ACCESSION NUMBERS

The rDNA sequences obtained from cDNA were deposited in the GenBank database. Accession numbers are KC663721–KC664150 for 16S rDNA and KC664151–KC664772 for 18S rDNA.

## RESULTS

### MACROSCOPIC OBSERVATIONS

**FIGURE 1:**
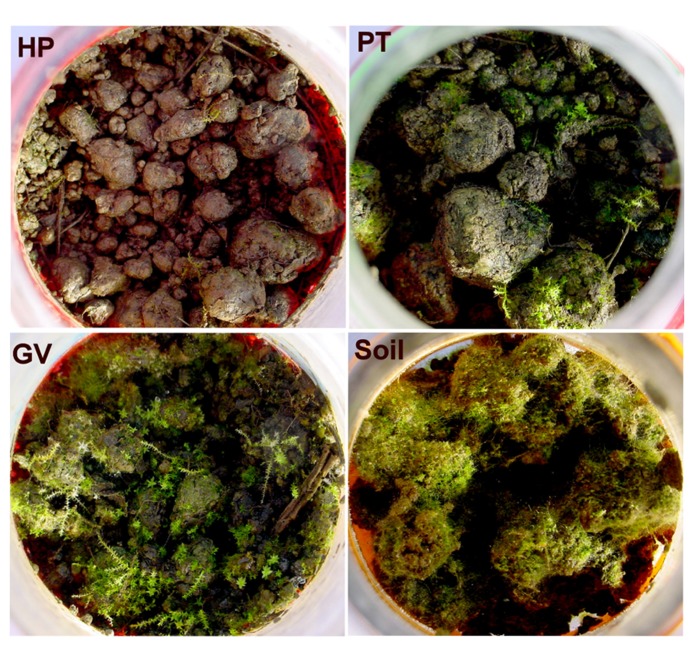
**Microcosm appearance after a 60-d incubation period: soil without added litter (lower right), soil with parental type litter (PT; upper right) litter, soil with litter from transformed poplars with a GUS vector (GV; lower left), and soil from transformed poplars with high proanthocyanidin content (HP; upper left)**.

At the end of the 60-d incubation, it became clear that moss preferentially proliferated in microcosms with lower tannin content (GV, PT, and litter-free controls), versus scant growth in microcosms with higher tannin content (HP). The mosses were identified from morphology and chloroplast rRNA genes as a mixture of *Ceratodon purpureus *(99% similarity), *Climacium americanum *(99% similarity), and a member of the Bryales (93% similarity to several species in the Bryales). Chloroplast sequences detected in the GV (KC663735, KC663739), PT (KC663842, KC663928), and HP (KC664033) soil samples corresponded to the moss taxa. Representative microcosms are shown in **Figure 1**. Moss coverage was visually estimated to be <1% for each HP soil sample (category 1), ca. 5% for each PT soil sample (category 2), ca. 50% for each GV soil samples (category 3), and total for each soil sample incubated without leaf litter (category 4).

### DENATURING GRADIENT GEL ELECTROPHORESIS

The results of the DGGE assay of 18S sequences showed a significant overall effect of litter type, incubation period, and their interaction (**Tables 2 and 3**). The number of distinct 18S sequences detected significantly increased with time for controls and all litter types except HP, where the number of bands remained the same. Microcosms with PT litter experienced a significantly (*P* < 0.000001, *F* = 27.3321, *df* = 3,24) greater increase, but the other litter types were not significantly different from controls. For DGGE of 16S rDNA, there was insufficient distinction between profuse numbers of diffuse bands to permit a clear comparative analysis.

**Table 2:** Summary of results for statistical tests used in analyses of variance for poplar litter experiments.

Experiment	Effect	*df*	*F*	*p*
18S DGGE^a^	Litter type	3,24	27.3321	<0.000001
	Incubation period	2,24	52.0689	<0.000001
	Interaction	6,24	9.0058	0.001370
16S rDNA	Treatment (N-fixer)	3,8	7.38679	0.010808
	Treatment (Nitrifier)	3,8	0.63665	0.612176
	Treatment (Cellulose degrader)	3,8	1.14348	0.388684
	Treatment (Phosphate solubilizer)	3,8	1.02327	0.432077

a A preliminary Levene’s test for these data indicated significant (*df* = 11, *F *= 6.127553, *p *= 0.000423) heterogeneity of variance which data transformations were unable to resolve. The analysis was therefore performed using a Weighted Least Squares method, with ANOVA weighted by inverse of the variance for each group.

**Table 3:** Mean number of 18S rDNA DGGE bands detected in DNA extracted from microcosm soils with poplar litter, after incubation for up to 60 d.

Litter type	Incubation period (d)	Mean number of bands
Parental type	0	2.3 ADIJ
	30	11.0 BEGH
	60	9.3 BCFGH
Gus Vector	0	1.0 AGIJ
	30	5.3 BCEFIJ
	60	6.0 BCFJ
High proanthocyanidin	0	3.0 ADEFIJ
	30	3.0 ADEFIJ
	60	3.0 ADFIJ
None	0	1.7 ADFIJ
	30	7.7 BCH
	60	6.7 BC

*Means in the same column followed by the same letter are not significantly different (*p * > 0.05) according to the Newman–Keuls multiple range test.*

### CLONED rDNA SEQUENCES – BACTERIAL DIVERSITY

**FIGURE 2:**
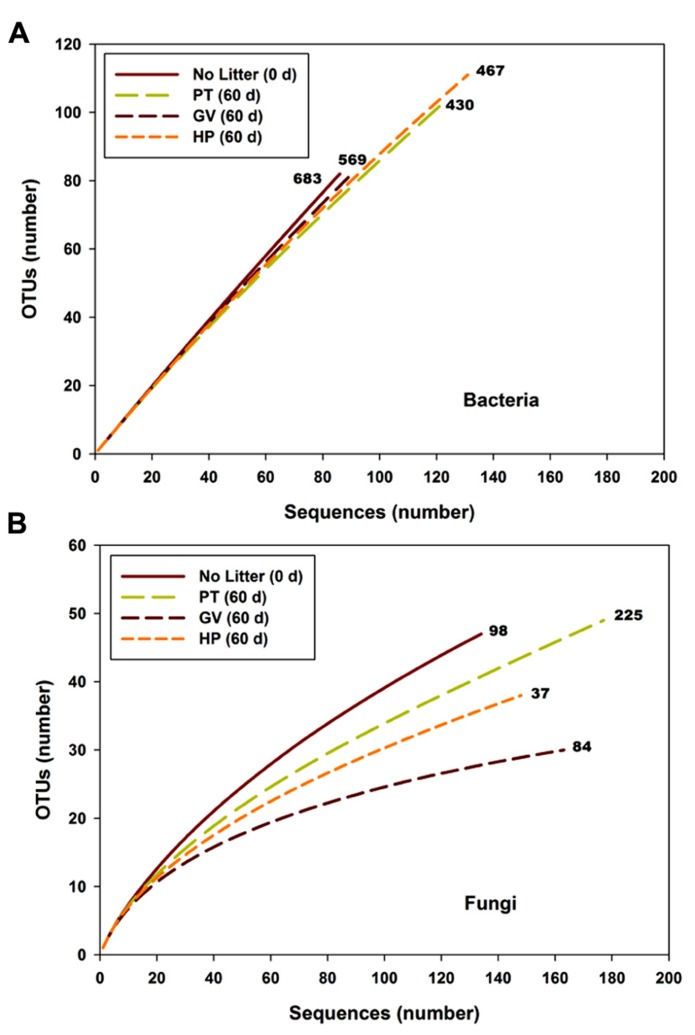
**Rarefaction curves for bacterial (A)** and fungal **(B)** sequences vs. OTUs found in microcosms with soil and leaf litter from *Populus tremuloides*. Curves are shown for soil before incubation (0 d) without added litter (Control), and soil from microcosms incubated for 60 d, with litter either from parental type (PT) saplings or transgenic poplar possessing either a simple GUS vector (GV) or higher expression of proanthocyanidins (HP). Numbers next to curves indicate the Chao richness estimate.

The slope of the sequence-based rarefaction curves was similar for all treatments (**Figure 2A**), indicating that soil bacterial diversity was comparable between all treatments. None of the rarefaction curves reached saturation, indicating that we did not capture all the bacterial diversity that was present. Overall, the proportion of Actinobacteria decreased between the control soil and all other treatments, whereas the proportion of Bacteroidetes, Chloroflexi, Cyanobacteria, and α-Proteobacteria significantly increased (**Table 4**). The only significant difference between the PT, GV, and HP treatments corresponded to β-Proteobacteria, which had proportionally higher numbers in HP and PT soils compared to GV soils (**Table 4**).

**Table 4:** Taxonomic classification of 16S rDNA sequences in microcosms with soil and leaf litter from *Populus tremuloides*.

	Mean proportion of sequences^a^			
	PT (60 d)	GV^b^ (60 d)	HP (60 d)	No litter^c^ (0 d)
Acidobacteria	23.4	19.1	13	19.8
Actinobacteria	3.2	9	9.2	24.4*
Bacteroidetes	2.4	3.4	3.8	0*
Chloroflexi	3.2	1.1	3.8	1.2*
Cyanobacteria	8.9	4.5	9.9	0*
Firmicutes	0.8	0	0	1.2
Gemmatimonadetes	1.6	1.1	1.5	2.3
Nitrospira	0	0	0	1.2
Planctomycetes	0	0	1.5	1.2
**Proteobacteria**				
α-Proteobacteria	25.8	32.6	26	15.1*
β-Proteobacteria	5.7	1.1*	9.9	9.1
δ-Proteobacteria	8.1	11.3	8.4	3.5
γ-Proteobacteria	3.2	4.5	3	3.5
Unclassified Proteobacteria	2.4	0	0.8	1.2
TM7	0	0	0	1.2
Unclassified	10.5	11.2	6.9	12.8
Verrucomicrobia	0.8	1.1	2.3	2.3

*PT, parental type litter (60 d); GV, GUS vector; HP, high proanthocyanidin content.*

a Abbreviated soil types are followed by days of incubation.

b The mean in this column marked with an asterisk (*) is significantly different (*P *= 0.002) than the mean for microcosms with other incubated soils (i.e., treatment affect corresponding to the mean of PT and HP soils in same row) according to Poisson linear regression analysis.

c Means in this column marked with an asterisk (*) are significantly different (*P *< 0.002) than the mean for microcosms sampled 60 d later (i.e., incubation effect corresponding to the mean of other soils in the same row) according to Poisson linear regression analysis.

The number of OTUs, Chao richness estimator and Shannon diversity index computed for each treatment are displayed in **Table 5**. The results indicate that neither richness nor diversity was significantly different between the treatments.

**Table 5:** Diversity of bacterial communities in microcosms with soil and leaf litter from *Populus tremuloides*.

Soil type^a^	Chao richness estimator	Shannon diversity index
PT (60 d)	569	4.55
GV (60 d)	467	4.36
HP (60 d)	430	4.65
No litter (0 d)	683	4.39

a Including days of incubation PT, parental type litter; GV, GUS vector, HP, high proanthocyanidin content.

**FIGURE 3:**
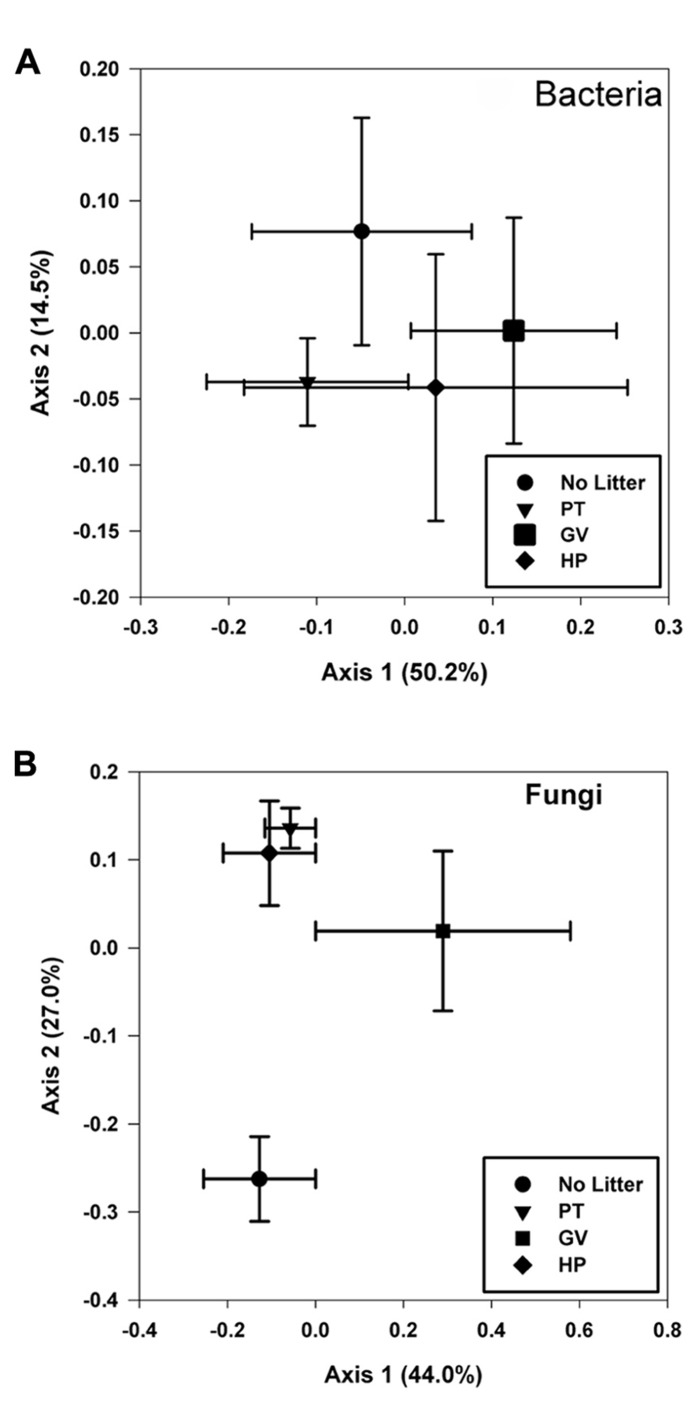
**Principal coordinate analyses of bacterial (A)** and fungal **(B)** communities found in microcosms with soil and leaf litter from *Populus tremuloides*. Communities correspond to soil in microcosms before incubation (0 d) and without added litter (Control), and soil from microcosms incubated for 60 d, with litter either from parental type (PT) saplings or transgenic poplar possessing either a simple GUS vector (GV) or higher expression of proanthocyanidins (HP).

The first two axis from the PCoA explained 64.7% of the variance and the results showed that none of the four treatments had a unique bacterial community, as they all clustered more or less together, indicating that there was no significant impact of any of the treatments on overall bacterial diversity (**Figure 3A**).

### CLONED rDNA SEQUENCES – DIVERSITY OF NUTRIENT CYCLING BACTERIAL GROUPS

Comparison of treatments in MANOVA produced a significant (*P *= 0.051, *F* = 7.38679, *df* = 3,8) difference in the proportion of known N-fixers (**Tables 2 and 6**). Isolated sequences corresponding to taxa that we presumed to be N-fixers included:* Aphanizomenon flos-aquae*, *Azospirillum* sp., *Bradyrhizobium elkanii*, * Bradyrhizobium iriomotense*, *Bradyrhizobium sp.*, *Burkholderia hospita*, *Calothrix* sp., *Mezorhizobium australicum*, *Nordella oligomobilis*, *Nostoc calcicola*, *Nostoc ellipsosporum*, *Phyllobacterium myrsinacearum*, *Rhodospirillaceae, *and *Sphingomonas* sp. MANOVA did not detect any significant difference between treatments versus the proportion of identifiable nitrifiers (17–21%), phosphate solubilizers (25–26%), or cellulose degraders (33–36%; **Tables 2 and 6**).

**Table 6:** The impact of litter types on the mean proportion of various microbial communities in soil microcosms.

Litter type^a^	N-fixers (%)^a^	Nitrifiers (%)^b^	Cellulose degraders (%)^c^	Phosphate solubilizers (%)^c^
PT (60 d)	5.5 BC	18.7	30.7	25.0
GV (60 d)	7.6 A	13.7	36.2	25.0
HP (60 d)	5.2 AC	21.0	32.6	26.1
None (0 d)	6.2 BC	16.8	34.1	25.4

*PT, parental type litter; GV, GUS vector; HP, high proanthocyanidin content.*

a Including days of incubation.

b Means in this column followed by the same letter are not significantly different (*p *> 0.05) according to the Newman–Keuls multiple range test.

c *F* test was not significant (*P *> 0.05).

### CLONED rDNA SEQUENCES – FUNGAL DIVERSITY

According to the sequence-based rarefaction curves (**Figure 2B**), the GV treatment had an almost saturated slope, whereas treatment control had the steepest one. This result suggests that even though the sampling effort was similar for all treatments, it did not capture soil fungal diversity to the same extent. At the phylum level, the proportion of Ascomycota/Basidiomycota increased between control and all other treatments, with Ascomycota representing 18% at control and between 30 and 40% for 60-d incubated treatments (**Table 7**). More specifically, at the class level, Agaricomycetes populations significantly decreased from 42% to 3–9%, whereas the proportion of Dothideomycetes, Pucciniomycetes, and Tremellomycetes significantly increased; from 13% to 23–36% for Dothideomycetes, from 0% to 0.6–4.7% for Pucciniomycetes, and from 17% to 39–47% for Tremellomycetes (**Table 7**). There were some significant differences between treatments after the 60-d incubation. Agaricomycetes and Sordariomycetes were significantly more abundant in PT soil than in any other treatment, whereas Pucciniomycetes were nearly in significantly higher proportion in HP soil (**Table 7**). The Pucciniomycete sequences closely (>95%) matched the moss parasite *Eocronartium muscicola*. **Table 8** presents the observed richness along with Chao, ACE, and Jackknife richness estimators, and Shannon and Simpson diversity indices for each treatment. Analysis of variance performed on richness estimators and diversity indices revealed no significant difference between treatments.

**Table 7:** Taxonomic classification of 18S sequences in microcosms with soil and leaf litter from *Populus tremuloides*.

	Mean proportion of sequences^a^			
	PT^b^ (60 d)	GV (60 d)	HP^c^ (60 d)	No litter^d^ (0 d)
Agaricomycetes	8.7*	3.7	3.4	41.9*
Basal fungal lineages	4.4	7.4	2.0	7.4
Chytridiomycetes	0.0	0.0	0.0	0.7
Dothideomycetes	25.7	22.7	35.6	13.2*
Eurotiomycetes	1.6	0.0	0.7	0.0
Orbiliomycetes	0.0	1.8	0.0	0.0
Pucciniomycetes	1.1	0.6	4.7*	0.0*
Sordariomycetes	13.1*	5.5	0.7	5.1
Tremellomycetes	38.8	45.4	47.0	16.9*
Unclassified fungi	2.3	1.2	0.0	2.2
Others				
Metazoa	4.3	9.2	2.7	8.1
Rhizaria	0.0	2.5	1.3	4.4
Viridiplantae	0.0	0.0	2.0	0.0
Summary				
Ascomycetes	40.4	30.1	36.9	18.4*
Basidiomycetes	48.6	49.7	55.0	58.8
Others	10.9	20.2	8.1	22.8

*PT, parental type litter (60d); GV, GUS vector; HP, high proanthocyanidin content.*

a Abbreviated soil types are followed by days of incubation.

b Means in this column marked with an asterisk (*) are significantly different (*P *< 0.038) than the mean for microcosms with other incubated soils (i.e., treatment effect corresponding to the mean of GV and HP soils in same row) according to Poisson linear regression analysis.

c Means in this column marked with an asterisk (*) are nearly significantly different (*P* = 0.051) than the mean for microcosms with other incubated soils (i.e., treatment effect corresponding to the mean of GV and PT soils in same row) according to Poisson linear regression analysis.

d Means in this column marked with an asterisk (*) are significantly different (*P *< 0.042) than the mean for microcosms sampled 60 d later (i.e., incubation effect corresponding to the mean of other soils in the same row) according to Poisson linear regression analysis.

**Table 8:** Diversity of fungal communities in microcosms with soil and leaf litter from *Populus tremuloides*.

Soil type^a^	Chao richness estimator	Shannon diversity index
PT (60 d)	225	2.99
GV (60 d)	37	2.67
HP (60 d)	84	2.84
No litter (0 d)	98	3.08

*PT, parental type litter (60 d); GV, GUS vector; HP, high proanthocyanidin content.*

a Including days of incubation.

Results obtained from the PCoA (**Figure 3B**), for which the first two axes explain 71.0% of the variance within the dataset, partitioned the fungal communities into three groups: one including those with higher levels of tannin in litter from both PT and HP treatments; another one from the control soil and a separate one from the GV treatment.

## DISCUSSION

We hypothesized that microbial communities associated with poplar litter would be significantly different, in terms of structure and abundance of predominant species or groups, when exposed to poplar litter with elevated tannin levels. This prediction was substantiated for some species and functional groups, but not in all cases for all treatments. For example, the diversity of fungi detected by DGGE increased when low-tannin litter was added to microcosms, but not for addition of high-tannin litter. We attribute this to the proliferation of fungal decomposers in litter with lower condensed tannins, and a lack of fungal growth in litter with higher tannin levels. In future studies, correlation of fungal DGGE assays with quantitative data from cloned rDNA sequences would help to delineate which species respond to the presence of litter. The PCoA results for fungi also showed that communities in microcosms with litter clustered separately from those without, with the communities in the GV treatment also clustering separately from litter with higher tannin levels. We attributed this to a distinction between the impacts of litter added as a nutritional substrate versus effects of inhibitory higher tannins within that substrate. The attenuation of some fungal rarefaction curves suggested that subtle differences in diversity could be detected with further sampling.

Considered in the context of prior studies, our findings reinforce the notion that impacts of genetic transformations on microbial communities of soils will vary, depending on the plant and the specific transgenes expressed in the plant. Oliver et al. (2008) found no significant effects on bacterial diversity and minor potential effects on the fungal community when assaying poplars over-expressing polyphenol oxidase. Hampp et al. (1996) found that transformation of poplars with hygromycin marker or indoleacetic acid biosynthetic genes did not affect the ability of *Amanita muscaria* to form mycorrhizae. Results similar to these examples were obtained in work with endochitinase-transformed white spruce; Stefani et al. (2010) did not detect any interference with ectendomycorrhizal *Wilcoxina* spp. and Lamarche et al. (2011) did not detect any significant impacts of transformation on fungal biomass or community structure in such trees. By contrast, a study of *Eucalyptus grandis* × *urophylla* transformed with the *nptII* (kanamycin resistance) gene, under control of the pea *Lhcb1-2* promoter, detected significant changes in α-Proteobacteria and *Methylobacterium* communities. The changes among treatments were comparable in scale to variability in wild-type populations, but some could be specifically attributed to effects of the transformation on soil communities (Andreote et al., 2009). Transformation of silver birch trees with various genes had an impact on the abundance of roots associated with the ectomycorrhizal (EM) fungus *Paxillus involutus*, through effects on root anatomy. However, no overall effect on the ability to form mycorrhizal relationships was observed (Pasonen et al., 2005; Seppänen et al., 2007).

Field studies have also produced varied results. A study of hybrid poplar (*Populus tremula* × *tremuloides*) with an altered phytohormone profile found no difference in EM colonization and diversity, although one transformed line produced changes in the abundances of some EM fungi (Kaldorf et al., 2002). Newhouse et al. (2007) found little effect of transformation with genes for an antimicrobial peptide on colonization rates for EM fungi. Stefani et al. (2009) found no long-term impacts on EM fungal diversity associated with field-deployed poplar transformed with *nptII* marker and GUS reporter genes. Field studies of Bt-transformed white spruce trees have produced variable results, depending on methodologies and the communities examined. LeBlanc et al. (2007) detected differences in 16S rRNA sequences extracted from soils associated with transformed spruce. However, Lamarche and Hamelin (2007) found no evidence for any impact of this transformation on rhizospheric diazotroph communities. In New Zealand field trials, some lines of *Pinus radiata* transformed with *LEAFY* and *nptII* genes produced ephemeral differences in some 16S rRNA profiles (α-Proteobacteria, Actinobacteria) detected with DGGE; however, overall effects on fungi and bacteria were judged to be insignificant (Lottman et al., 2010).

The proportion of N-fixing bacteria was significantly greater in microcosms with low-tannin litter in our study. We attribute this to the presence of cellulosic substrate and nutrients available from decomposition. Several of the genera detected (*Burkholderia*, *Sphingomonas*) are reported to be diazotrophic endophytes of *Populus trichocarpa* and willow (*Salix sitchensis*) native to the Pacific Northwest (Doty et al., 2009). There was no such increase when added litter contained higher amounts of tannin; the proportion of N-fixing bacteria remained similar to the lower proportion at the start of incubation. We attribute this result to an inhibitory effect of proanthocyanidins at higher (parental or transgenic) concentrations; phenolics, including tannins, were reported to inhibit N-fixation in a study of decomposing litter from mangroves (Pelegrí and Twilley, 1998). It should be noted that an increase in the occurrence of N-fixing organisms relates to the potential for N-fixation. Our experiment detected the presence of N-fixing organisms, but did not differentiate DNA from active vs. non-active N-fixers. Our assumption is that abundance reflects functional behavior in this instance, but it should nevertheless be noted that actual functional behavior might not only directly reflect changes in overall abundance. Further experimentation, e.g., with mRNA or N assays, would be required to confirm functional impacts.

Impacts of litter on the proportion of nitrifiers were negligible. However, observed contrasts only cover an incubation period of 60 d; ephemeral differences occurring before that point would not be captured in this analysis. Likewise, later differences due to differing decomposition rates (Preston et al., 2009a, b) or persistent inhibitory effects could also manifest. Increased sampling for a longer period would be needed to address these possibilities. While no significant changes in populations of nitrifiers, cellulose degraders, or phosphate solubilizers were detected, there is a possibility that minor changes could be detected with more extensive replication more precise identification, and targeting of other taxa within functional groups. For example, the study did not include a focus on archaea, and yet they may be dominant and more responsive in soils with low N (Leininger et al., 2006; Nicol et al., 2008; Huang et al., 2012; Zhalnina et al., 2012). Moreover, the functional response of microbial communities may be poorly reflected by population levels, or confounded by other functional responses or factors. *Acidobacteria* spp., for example, may possess the capacity to utilize cellulose and to reduce nitrates and nitrates (reversing nitrification) where soil nutrients are relatively low and soil moisture varies (Ward et al., 2009). They may also produce acid phosphatases (Koch et al., 2008) and dominate in soil zones where there is intense phosphate mobilization (Green et al., 2002). Factors such as soil carbon and pH may also affect *Acidobacteria* communities (Koch et al., 2008; Fierer et al., 2013). For overall functional impacts, shifts of species composing a functional group may therefore be as important as changes in numbers.

The impacts of proanthocyanidins on the bacterial communities observed here may have resulted from direct affects on bacterial growth, or indirect effects related to moss growth. For example, Bragina et al. (2012) have shown that patterns of α-Proteobacteria and other bacterial taxa are affected by moss species. Effects of proanthocyanidins on moss growth would therefore also be likely to also have an impact on the community structure of microcosm soil bacteria.

DNA associated with Pucciniomycetes increased when litter was added, and most closely matched *Eocronartium muscicola*, a parasite of mosses (Aime et al., 2006). Boehm and McLaughlin (1988) reported that *Eocronartium muscicola* can arrest or supplant sporophyte growth. We attribute the growth of moss in the microcosms to the likely presence of moss protonema cells in the soils, before litter addition. The nearly significant increase of a putative moss parasite in litter with higher tannin levels suggests that the fungus may have been involved with the reduced moss growth shown in **Figure 1**. Further experimentation with these microcosms would be needed to understand how soil tannins might promote proliferation of the parasite.

The results show that condensed tannin content of litter may have implications for the rate of decomposition and soil nutrient cycling processes. Both natural genotypic variation and environmental factors influence tannin production, causing proanthocyanidin content to vary in natural poplar populations (Mansfield et al., 1999; Lindroth et al., 2002; Constabel and Lindroth, 2010). Even the highest tannin levels found in this experiment fell within the natural range of variation observed in forests (Preston et al., 2009a, b). The alteration in tannin levels due to the MYB 134 over-expression was thus in line with what could be expected from natural variation in proanthocyanidin content, and also with expected *in situ* variability. For the specific trait manipulated, i.e., tannin content, there would therefore be no environmental risks above and beyond those that would accompany conventional tree breeding efforts for this trait. Our work also found no evidence that there are additional environmental risks of the genetic transformation process itself for the soil microbe environment. It is important to note, however, that manipulation of specific traits, even if very targeted, can sometimes have unexpected secondary effects. For example, in the case of the MYB134 over-expressing plants, high-tannin levels are reported to be accompanied by reduced salicinoid levels, likely due to internal metabolic trade-offs (Mellway et al., 2009). A full exploration of the effects of these phenolic antiherbivory compounds and other interrelated effects on soil microbes are beyond the scope of this work, we cannot rule out that at least some of the effects in the HP treatment might also be due to variable salicinoids or other factors.

The microcosms used in this study only had incorporated soil. In further microcosm studies, it would be interesting to incorporate tree seedlings, understorey plants, EM fungi, earthworms, soil fauna, etc., to improve comparison to natural (*in situ*) conditions. A complete examination of how nutrient cycling phenomena work with respect to high-tannin litters is an important question for future study. The moss growing in these experiments likely had some impact via N uptake and immobilization (Weber and Van Cleve, 1984), fulfilling the role of understorey plants to some degree. Expanding the analyses to include sequences from incubated litter-free soil and incremental tannin measurements throughout the incubation period would also help to characterize responses. In future trials, improved risk assessments might also determine any effect of sterile vs. non-sterile litter, and include soils from plantations of the INRA 353-38 *Populus tremula* × *tremuloides* hybrid and/or variety of *Populus* spp. and hybrids.

The approach used in this study had the advantage of requiring fewer resources than, for example, 454 pyrosequencing or other next-generation sequencing methods. The use of 454 pyrosequencing could improve detection of some of the more subtle influences of litter on the soil communities, for example details on the occurrence of rare types. It could potentially help to clarify the observed differences in rarefaction rates for the 18S signal, for example. However, the approach used in this study had the advantage of requiring fewer resources and avoiding saturation of the sequence dataset. DGGE, rDNA cloning and sequencing used here were sufficient to detect microbial dynamics in the microcosms. For nutrient cycling and other functions, the population dynamics of key, dominant species are likely the most relevant, unless a significant functional role can be demonstrated for the rare types.

Potential impacts (and therefore potential risks) associated with transgenic trees have been highlighted in various reports, but there are also potential risks in failing to respond to wide-scale anthropogenic impacts on forests. These may whether take the form of potential extirpations from recent pest introductions, or emerging threats such as climate change. Safe modification of tree traits offers the opportunity to streamline ongoing tree-breeding efforts to counter these threats. There is therefore a need to develop methods to assess the safety of genetically modified trees, even in areas where their commercial use is currently in question (McLean and Charest, 2000). Our work indicates that impacts of transgenic leaf litter on key forest soil communities can be effectively and safely evaluated in microcosms as a first step in characterizing the potential impacts (or lack thereof) that might be associated with a particular genetic modification. It also demonstrates the value of genetic transformations and microcosm research in exploring the genetic basis for ecological phenomena such as tannin impacts on soils.

## Conflict of Interest Statement

The authors declare that the research was conducted in the absence of any commercial or financial relationships that could be construed as a potential conflict of interest.

## References

[B1] Adamczyk B. Kitunen V. Smolander A. (2009). Polyphenol oxidase, tannase and proteolytic activity in relation to tannin concentration in the soil organic horizon under silver birch and Norway spruce. *Soil Biol. Biochem.* 5 2085–2093 10.1016/j.soilbio.2009.07.018

[B2] Aime M. C. Matheny P. B. Henk D. Frieders E. M. Nilsson R. H. Piepenbring M. (2006). An overview of the higher level classification of Pucciniomycotina based on combined analyses of nuclear large and small subunit rDNA sequences. *Mycologia* 98 896–905 10.3852/mycologia.98.6.896 17486966

[B3] Altschul S. F. Gish W. Miller W. Myers E. W. Lipman D. J. (1990). Basic local alignment search tool. *J. Mol. Biol.* 215 403–410 10.1016/S0022-2836(05)80360-2 2231712

[B4] Andreote F. D. Carneiro R. T. Salles J. F. Marcon J. Labate C. A. Azevedo J. L. (2009). Culture-independent assessment of Rhizobiales-related Alphaproteobacteria and the diversity of Methylobacterium in the rhizosphere and rhizoplane of transgenic eucalyptus. *Microb. Ecol.* 57 82–93 10.1007/s00248-008-9405-8 18536862

[B5] Anonymous (2001). *Qiagen PCR Cloning Handbook for Qiagen PCR Cloning Kit.* Mississauga, ON: Qiagen, Inc

[B6] Boehm E. W. A. McLaughlin D. J. (1988). *Eocronartium muscicola*: a basidiomycetous moss parasite exploiting gametophytic transfer cells. *Can. J. Bot.* 66 762–770 10.1139/b88-113

[B7] Bolton H. Fredrickson J. K. Bentjen S. A. Workman D. J. Li S. W. Thomas J. M. (1991). Field calibration of soil-core microcosms: fate of a genetically altered Rhizobacterium. *Microb. Ecol.* 21 163–173 10.1007/BF02539151 24194208

[B8] Borneman J. Hartin R. J. (2000). PCR primers that amplify fungal rRNA genes from environmental samples. *Appl. Environ. Microbiol.* 66 4356–4436 10.1128/AEM.66.10.4356-4360.2000 11010882 PMC92308

[B9] Bradley R. L. Titus B. D. Preston C. M. (2000). Changes to mineral N cycling and microbial communities in black spruce humus after additions of (NH4)2SO4 and condensed tannins extracted from *Kalmia angustifolia* and balsam fir. *Soil Biol. Biochem.* 32 1277–1240 10.1016/S0038-0717(00)00039-0

[B10] Bragina A. Berg C. Cardinale M. Shcherbakov A. Chebotar V. Berg G. (2012). Sphagnum mosses harbour highly specific bacterial diversity during their whole lifecycle. *ISME J.* 6 802–813 10.1038/ismej.2011.151 22094342 PMC3309359

[B11] Constabel C. P. Lindroth R. I. (2010). ``The impact of genomics on advances in herbivore defense and secondary metabolism in *Populus*,'' in *Genetics and Genomics of Populus* eds Jannson S. Bhaleaero R. Grover A. (New York: Spinger Verlag) 279–305

[B12] Doty S. L. Oakley B. Xin G. Kang J. W. Singleton G. Khan Z. (2009). Diazotrophic endophytes of native black cottonwood and willow. *Symbiosis* 47 23–33 10.1007/BF03179967

[B13] Edgar R. C. (2004). MUSCLE: multiple sequence alignment with high accuracy and high throughput. *Nucleic Acids Res.* 32 1792–1797 10.1093/nar/gkh340 15034147 PMC390337

[B14] Fierer N. Bradford M. A. Jackson R. B. (2013). Toward an ecological classification of soil bacteria. *Ecology* 88 1354–1364 10.1890/05-1839 17601128

[B15] Fierer N. Schimel J. P. Cates R. G. Zou J. (2001). Influence of balsam poplar tannin fractions on carbon and nitrogen dynamics in Alaskan taiga floodplain soils. *Soil Biol. Biochem.* 33 1827–1839 10.1016/S0038-0717(01)00111-0

[B16] Fortin N. Beaumier K. L. Greer C. W. (2004). Soil washing improves the recovery of total community DNA from polluted and high organic content sediments. *J. Microbiol. Methods* 56 181–191 10.1016/j.mimet.2003.10.006 14744447

[B17] Gartland K. M. A. Crow R. M. Fenning T. M. Gartland J. S. (2003). Genetically modified trees: production, properties, and potential. *Arboric. Urban For. (J. Arboricult.)* 29 259–266

[B18] Green E. G. Macalady J. L. Banfield J. F. (2002). Biogeochemical contributions to soil formation and landscape lowering. *AGU Fall Meet. Abs. #H* 12B-0929

[B19] Halpin C. Thain S. C. Tilston E. L. Guiney E. Lapierre C. Hopkins D. W. (2007). Ecological impacts of trees with modified lignin. *Tree Genet. Genomes* 3 101–110 10.1007/s11295-006-0060-2

[B20] Hampp R. Ecke M. Schaeffer C. Wallenda T. Wingler A. Kottke I. (1996). Axenic mycorrhization of wild type and transgenic hybrid aspen expressing T-DNA indoleacetic acid-biosynthetic genes. *Trees* 11 59–64 10.1007/s004680050059

[B21] Hay I. Morency M. Seguin A. (2002). Assessing the persistence of DNA in decomposing leaves of genetically modified poplar trees. *Can. J. For. Res.* 32 977–982 10.1139/x02-017

[B22] Holeski L. Vogelzang A. Stanosz G. Lindroth R. L. (2009). Incidence of *Venturia* shoot blight in aspen (*Populus tremuloides* Michx.)varies with tree chemistry and genotype. *Biochem. Syst. Ecol.* 37 139–145 10.1016/j.bse.2009.02.003

[B23] Howell A. B. Vorsa N. Der Marderosian A. Foo L. Y. (1998). Inhibition of the adherence of P-fimbriated *Escherichia coli* to uroepithelial-cell surfaces by proanthocyanidin extracts from cranberries. *N. Eng. J. Med.* 339 1085–1086 10.1056/NEJM199810083391516 9767006

[B24] Huang R. Wu Y. Zhang J. Zhong W. Jia Z. Cai Z. (2012). Nitrification activity and putative ammonia-oxidizing archaea in acidic red soils. *J. Soils Sediments* 12 420–428 10.1007/s11368-011-0450-4

[B25] Huber T. Faulkner G. Hugenholtz P. (2004). Bellerophon: a program to detect chimeric sequences in multiple sequence alignments. *Bioinformatics* 20 2317–2319 10.1093/bioinformatics/bth226 15073015

[B26] Jansson S. Douglas C. J. (2007). *Populus*: a model system for plant biology. *Annu. Rev. Plant Biol.* 58 435–458 10.1146/annurev.arplant.58.032806.103956 17280524

[B27] Joanisse G. D. Bradley R. L. Preston C. M. Munson A. D. (2007). Soil enzyme inhibition by condensed litter tannins may drive ecosystem structure and processes: the case of *Kalmia angustifolia*. *New Phytol.* 175 535–546 10.1111/j.1469-8137.2007.02113.x 17635228

[B28] Kaldorf M. Fladung M. Muhs H.-J. Buscot F. (2002). Mycorrhizal colonization of transgenic aspen in a field trial. *Planta* 214 653–660 10.1007/s004250100658 11925050

[B29] Kelley C. C. Spilsbury R. H. (1939). *Soil survey of the Lower Fraser Valley*. Dominion of Canada Dept. of Agriculture Ottawa Publ. 650, Tech. Bull. 20.

[B30] Koch I. H. Gich F. Dunfield P. F. Overmann J. (2008). *Edaphobacter modestus* gen. nov., sp. nov., and *Edaphobacter aggregans* sp. nov., acidobacteria isolated from alpine and forest soils. *Int. J. Syst. Evol. Microbiol.* 58 1114–1122 10.1099/ijs.0.65303-0 18450699

[B31] Kraus T. E. Zazoski R. J. Dahlgren R. A. Horwath W. R. Preston C. M. (2004). Carbon and nitrogen dynamics in a forest soil amended with purified tannins from different plant species. *Soil Biol. Biochem.* 36 309–321 10.1016/j.soilbio.2003.10.006

[B32] Krimsky S. Wrubel R. P. Naess I. G. Levy S. B. Wetzler R. E. Marshall B. (1995). Standardized microcosms in microbial risk assessment. *BioScience* 45 590–599 10.2307/1312763

[B33] Lamarche J. Hamelin R. C. (2007). No evidence of impact of Bt-transgenic white spruce expressing the CrylAb toxin of *Bacillus thuringiensis* on rhizosphere diazotroph community. *Appl. Environ. Microbiol.* 73 6577–6583 10.1128/AEM.00812-07 17660307 PMC2075053

[B34] Lamarche J. Stefani F. O. P. Séguin A. Hamelin R. C. (2011). Impact of endochitinase-trasformed white spruce on soil fungal communities under greenhouse conditions. *FEMS Microbiol. Ecol.* 76 199–208 10.1111/j.1574-6941.2011.01041.x 21223334

[B35] LeBlanc P. M. Hamelin R. C. Filion M. (2007). Alteration of soil rhizosphere communities following genetic transformation of white spruce. *Appl. Environ. Microbiol.* 73 4128–4134 10.1128/AEM.02590-06 17468272 PMC1932765

[B36] Leininger S. Urich T. Schloter M. Schwark L. Qi J. Nicol G. W. (2006). Archaea predominate among ammonia-oxidizing prokaryotes in soils. *Nature* 442 806–809 10.1038/nature04983 16915287

[B37] Leple J. C. Bonade-Bottino M. Augustin S. Pilate G. Dumanois Le Tan V. Delplanque A. (1995). Toxicity to *Chrysomela tremulae* (Coleoptera: Chrysomelidae) of transgenic poplars expressing a cysteine proteinase inhibitor. *Mol. Breed.* 1 319–328 10.1007/BF01248409

[B38] Lindroth R. L. Osier T. L. Barnhill H. R. H. Wood S. A. (2002). Effects of genotype and nutrient availability on phytochemistry of trembling aspen (*Populus tremuloides* Michx.) during leaf senescence. *Biochem. Syst. Ecol.* 30 297–307 10.1016/S0305-1978(01)00088-6

[B39] Lottman J. O'Callaghan M. Barid D. Walter C. (2010). Bacterial and fungal communities in the rhizosphere of field-grown genetically modified pine trees (*Pinus radiata* D.). *Environ. Biosafety Res.* 9 25–40 10.1051/ebr/2010007 21122484

[B40] Lozupone C. Knight R. (2005). UniFrac: a new phylogenetic method for comparing microbial communities. *Appl. Environ. Microbiol.* 71 8228–8235 10.1128/AEM.71.12.8228-8235.2005 16332807 PMC1317376

[B41] Machinet G. E. Betrand I. Chabbert B. Recous S. (2009). Decomposition in soil and chemical changes of maize roots with genetic variations affecting cell wall quality. *Eur. J. Soil Sci.* 60 176–185 10.1111/j.1365-2389.2008.01109.x

[B42] Madritch M. Donaldson J. Lindroth R. (2006). Genetic identity of *Populus tremuloides* litter influences decomposition and nutrient release in a mixed forest stand. *Ecosystems* 9 528–537 10.1007/s10021-006-0008-2

[B43] Madritch M. Hunter M. (2003). Intraspecific litter diversity and nitrogen deposition affect nutrient dynamics and soil respiration. *Oecologia* 136 124–128 10.1007/s00442-003-1253-0 12684853

[B44] Mansfield J. (1999). Genotypic variation for condense tannin production in trembling aspen. *Am. J. Bot.* 86 1154–1159 10.2307/2656979 10449395

[B45] Mansfield J. L. Curtis P. S. Zak D. R. Pregitzer K. S. (1999). Genotypic variation for condensed tannin production in trembling aspen (*Populus tremuloides*, Salicaceae) under elevated CO2 and in high- and low-fertility soil. *Am. J. Bot.* 86 1154–1159 10.2307/2656979 10449395

[B46] Marvier M. (2002). Improving risk assessment for nontarget safety of transgenic crops. *Ecol. Appl.* 12 1119–1124 10.1890/1051-0761(2002)012[1119:IRAFNS]2.0.CO;2

[B47] McGuire K. L. Treseder K. K. (2009). Microbial communities and their relevance for ecosystem models: decomposition as a case study. *Soil Biol. Biochem.* 42 529–535 10.1016/j.soilbio.2009.11.016

[B48] McLean M. A. Charest P. J. (2000). The regulation of transgenic trees in North America. *Silvae Genet.* 49 233–239

[B49] Mellway R. D. Tran L. T. Prouse M. B. Campbell M. M. Constabel C. P. (2009). The wound-, pathogen-, and ultraviolet B-responsive MYB134 gene encodes an R2R3 MYB transcription factor that regulates proanthocyanidin synthesis in poplar. *Plant Physiol.* 150 924–941 10.1104/pp.109.139071 19395405 PMC2689947

[B50] Miranda M. Ralph S. G. Mellway R. White R. Heath M. C. Bohlmann J. (2007). The transcriptional response of hybrid poplar (*Populus trichocarpa* x *P. deltoides*) to infection by *Melampsora medusae* leaf rust involves induction of flavonoid pathway genes leading to the accumulation of proanthocyanidins. *Mol. Plant Microbe Int.* 20 816–831 10.1094/MPMI-20-7-0816 17601169

[B51] Morse A. M. Cooke J. E. K. Davis J. M. (2004). ``Forest tree functional genomics,'' in *Molecular Genetics and Breeding of Forest Trees* eds. Kumar S. Fladung M. (Binghamton: Haworth Press) 3–17

[B52] Newhouse A. E. Schrodt F. Liang H. Maynard C. A. Powell W. A. (2007). Transgenic American elm shows reduced Dutch elm disease symptoms and normal mycorrhizal colonization. *Plant Cell Rep.* 26 977–987 10.1007/s00299-007-0313-z 17310333

[B53] Nicol G. W. Leininger S. Schleper C. Prosser J. I. (2008). The influence of soil pH on the diversity, abundance and transcriptional activity of ammonia oxidizing archaea and bacteria. *Environ. Microbiol.* 10 2965–2978 10.1111/j.1462-2920.2008.01701.x 18707610

[B54] Norris C. E. Preston C. M. Hogg K. E. Titus B. D. (2011). The influence of condensed tannin structure on rate of microbial mineralization and reactivity to chemical assays. *J. Chem. Ecol.* 37 311–319 10.1007/s10886-011-9921-8 21340461

[B55] Oliver K. L. Hamelin R. C. Hintz W. E. (2008). Effects of transgenic hybrid aspen overexpressing polyphenol oxidase on rhizosphere diversity. *Appl. Environ. Microbiol.* 74 5340–5348 10.1128/AEM.02836-07 18552195 PMC2546652

[B56] Pasonen H.-L. Degefu Y. Brumós J. Lohtander K. Pappinen A. Timonen S. (2005). Transgenic *Betula pendula* expressing sugar beet chitinase IV forms normal ectomycorrhizae with *Paxillus involutus* in vitro. *Scand. J. For. Res.* 20 385–392 10.1080/02827580500251432

[B57] Pelegrï S. P. Twilley R. R. (1998). Heterotrophic nitrogen fixation (acetylene reduction) during leaf-litter decomposition of two mangrove species from South Florida, USA. *Mar. Biol.* 131 53–61 10.1007/s002270050296

[B58] Peña L. Seguin A. (2001). Recent advances in the genetic transformation of trees. *Trends Biotechnol.* 19 500–506 10.1016/S0167-7799(01)01815-7 11711193

[B59] Porter L. J. Hrstich L. N. Chan B. G. (1986). The conversion of procyanidins and prodelphinidins to cyanidin and delphinidin. *Phytochemistry* 25 223–230 10.1016/S0031-9422(00)94533-3

[B60] Preston C. M. Nault J. R. Trofymow J. A. (2009a). Chemical changes during 6 years of decomposition of 11 litters in some Canadian forest sites. Part 2. 13C Abundance, Solid-State 13C NMR Spectroscopy and the Meaning of ``Lignin''. *Ecosystems* 12 1078–1102 10.1007/s10021-009-9267-z

[B61] Preston C. M. Nault J. R. Trofymow J. A. Smyth C. CIDET Working Group (2009b). Chemical changes during 6 years of decomposition of 11 litters in some Canadian forest sites. Part 1. Elemental composition, tannins, phenolics, and proximate fractions. *Ecosystems* 12 1053–1077 10.1007/s10021-009-9266-0

[B62] Quinsey S. Stettler R. F. Heilman P. E. Delany D. Fenn R. Ager A. (1991). *Pedigree Clone Register*. University of Washington/Washington State University Poplar Research Program. College of Forest Resources, University of Washington Seattle

[B63] R Development Core Team. (2005). *R: A Language and Environment for Statistical Computing*. Vienna: R Foundation for Statistical Computing

[B64] Scalbert A. (1991). Antimicrobial properties of tannins. *Phytochemistry* 30 3875–3883 10.1016/0031-9422(91)83426-L

[B65] Schimel J. P. Cates R. G. Ruess R. (1998). The role of balsam poplar secondary chemicals in controlling soil nutrient dynamics through succession in the Alaska taiga. *Biogeochemistry* 42 221–234 10.1023/A:1005911118982

[B66] Schloss P. D. Westcott S. L. Ryabin T. Hall J. R. Hartmann M. Hollister E. B. (2009). Introducing mothur: open-source, platform-independent, community-supported software for describing and comparing microbial communities. *Appl. Environ. Microbiol.* 75 7537–7541 10.1128/AEM.01541-09 19801464 PMC2786419

[B67] Schweitzer J. A. Bailey J. K. Fischer D. G. LeRoy C. J. Lonsdorf E. V. Whitham T. G. (2008). Plant–soil–microorganism interactions: heritable relationship between plant genotype and associated soil microorganisms. *Ecology* 89 773–781 10.1890/07-0337.1 18459340

[B68] Schweitzer J. A. Bailey J. K. Rehill B. J. Martinsen G. D. Hart S. C. Lindroth R. L. (2004). Genetically based trait in a dominant tree affects ecosystem processes. *Ecol. Lett.* 7 127–134 10.1111/j.1461-0248.2003.00562.x

[B69] Seppänen S.-K. Pasonen H.-L. Vauramo S. Vahala J. Toikka M. Kilpeläinen I. (2007). Decomposition of the leaf litter and mycorrhiza forming ability of silver birch with a genetically modified lignin biosynthesis pathway. *Appl. Soil Ecol.* 36 100–106 10.1016/j.apsoil.2006.12.002

[B70] Stanton B. Eaton J. Johnson J. Rice D. Schuette B. Moser B. (2002). Hybrid poplar in the Pacific Northwest. The effects of market-driven management. *J. For.* 100 28–33

[B71] Stefani F. O. P. Mancalvo J.-M. Séguin A. Bérubé J. A. Hamelin R. C. (2009). Impact of an 8-year-old transgenic poplar plantation on the ectomycorrhizal fungal community. *Appl. Environ. Microbiol.* 75 7527–7536 10.1128/AEM.01120-09 19801471 PMC2786396

[B72] Stefani F. O. P. Tanguay P. Pelletier G. Piché Y. Hamelin R. C. (2010). Impact of endochitinase-transformed white spruce on soil fungal biomass and ectendomycorrhizal symbiosis. *Appl. Envrion. Microbiol.* 76 2607–2614 10.1128/AEM.02807-09 PMC2849194 20173071

[B73] Tamura K. Dudley J. Nei M. Kumar S. (2007). MEGA4: Molecular Evolutionary Genetics Analysis (MEGA) software version 4.0. *Mol. Biol. Evol.* 24 1596–1599 10.1093/molbev/msm092 17488738

[B74] Teuben A. Verhoef H. A. (1992). Direct contribution by soil arthropods to nutrient availability through body and faecal nutrient content. *Biol. Fertil. Soils* 14 71–75 10.1007/BF00336253

[B75] Triebwasser D. J. Tharayil N. Preston C. M. Gerard P. D. (2012). The susceptibility of soil enzymes to inhibition by leaf litter tannins is dependent on the tannin chemistry, enzyme class and vegetation history. *New Phytol.* 196 1122–1132 10.1111/j.1469-8137.2012.04346.x 23025512

[B76] Vainio E. J. Hallaksela A. M. Lippone K. Hantula J. (2005). Direct analysis of ribosomal DNA in denaturing gradients: application on the effects of *Phlebiopsis gigantea* treatment on fungal communities of conifer stumps. *Mycol. Res.* 109 103–114 10.1017/S0953756204001406 15736868

[B77] van Frankenhuyzen K. Beardmore T. (2004). Current status and environmental impact of transgenic forest trees. *Can. J. For. Res.* 34 1163–1180 10.1139/x04-024

[B78] Vauramo S. Pasonen H. L. Pappinen A. Setala H. (2006). Decomposition of leaf litter from chitinase transgenic silver birch (*Betula pendula*) and effects on decomposer populations in a field trial. *Appl. Soil Ecol.* 32 338–349 10.1016/j.apsoil.2005.07.007

[B79] Wang Q. Garrity G. M. Tiedje J. M. Cole J. R. (2007). Naïve Bayesian classifier for rapid assignment of rRNA sequences into the new bacterial taxonomy. *Appl. Environ. Microbiol.* 73 5261–5267 10.1128/AEM.00062-07 17586664 PMC1950982

[B80] Ward N. L. Challacombe J. F. Janssen P. H. Henrissat B. Coutinho P. M. Wu M. (2009). Three genomes from the phylum Acidobacteria provide insight into the lifestyles of these microorganisms in soils. *Appl. Environ. Microbiol.* 75 2046–2056 10.1128/AEM.02294-08 19201974 PMC2663196

[B81] Weber M. G Van Cleve K. (1984). Nitrogen transformations in feather moss and forest floor layers of interior Alaska black spruce ecosystems. *Can. J. For. Res.* 14 278–290 10.1139/x84-053

[B82] Witham T. G. Bailey J. K. Schweitzer J. A. Shuster S. M. Bangert R. K. LeRoy C. J. (2006). A framework for community and ecosystem genetics: from genes to ecosystems. *Nat. Rev. Genet.* 7 510–523 10.1038/nrg1877 16778835

[B83] Zhalnina K. de Quadros P. D. Carmargo F. A. O. Triplett E. W. (2012). Drivers of archaeal ammonia-oxidizing communities in soil. *Front. Microbiol.* 3:210 10.3389/fmicb.2012.00210 PMC3375578 22715335

